# Comparison of estimated GFR using cystatin C versus creatinine in pediatric kidney transplant recipients

**DOI:** 10.1007/s00467-024-06316-6

**Published:** 2024-03-01

**Authors:** Helen Pizzo, John Nguyen, George J. Schwartz, Katherine Wesseling-Perry, Robert Ettenger, Eileen Tsai Chambers, Patricia Weng

**Affiliations:** 1 Department of Pediatrics, Cedars-Sinai Medical Center, 8700 Beverly Blvd., Los Angeles, CA 90048 USA; 2 Children’s Hospital of Orange County, Orange, CA USA; 3 University of Rochester Medical Center, Rochester, NY USA; 4 Phoenix Children’s Hospital, Phoenix, AZ USA; 5 University of Arizona College of Medicine, Phoenix, AZ USA; 6 David Geffen School of Medicine at UCLA, Los Angeles, CA USA; 7 Duke University School of Medicine, Durham, NC USA

**Keywords:** Kidney function, Estimating equations, Bias, Accuracy, Precision

## Abstract

**Background:**

An accurate, rapid estimate of glomerular filtration rate (GFR) in kidney transplant patients affords early detection of transplant deterioration and timely intervention. This study compared the performance of serum creatinine (Cr) and cystatin C (CysC)-based GFR equations to measured GFR (mGFR) using iohexol among pediatric kidney transplant recipients.

**Methods:**

CysC, Cr, and mGFR were obtained from 45 kidney transplant patients, 1–18 years old. Cr- and CysC-estimated GFR (eGFR) was compared against mGFR using the Cr-based (Bedside Schwartz, U25-Cr), CysC-based (Gentian CysC, CAPA, U25-CysC), and Cr-CysC combination (CKiD Cr-CysC, U25 Cr-CysC) equations in terms of bias, precision, and accuracy. Bland–Altman plots assessed the agreement between eGFR and mGFR. Secondary analyses evaluated the formulas in patients with biopsy-proven histological changes, and K/DOQI CKD staging.

**Results:**

Bias was small with Gentian CysC (0.1 ml/min/1.73 m^2^); 88.9% and 37.8% of U25-CysC estimations were within 30% and 10% of mGFR, respectively. In subjects with histological changes on biopsy, Gentian CysC had a small bias and U25-CysC were more accurate—both with 83.3% of and 41.7% of estimates within 30% and 10% mGFR, respectively. Precision was better with U25-CysC, CKiD Cr-CysC, and U25 Cr-CysC. Bland–Altman plots showed the Bedside Schwartz, Gentian CysC, CAPA, and U25-CysC tend to overestimate GFR when > 100 ml/min/1.72 m^2^. CAPA misclassified CKD stage the least (whole cohort 24.4%, histological changes on biopsy 33.3%).

**Conclusions:**

In this small cohort, CysC-based equations with or without Cr may have better bias, precision, and accuracy in predicting GFR.

**Graphical abstract:**

**Figure:**
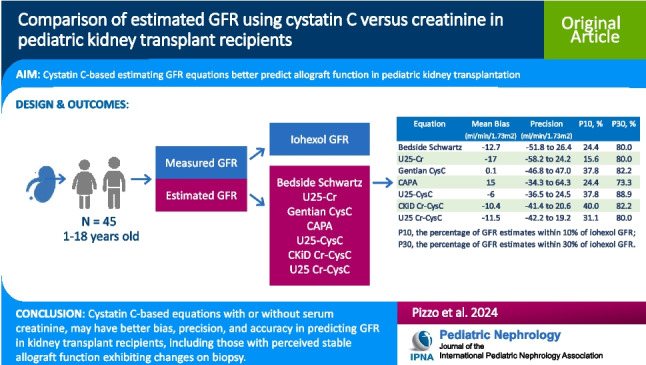
A higher resolution version of the Graphical abstract is available as Supplementary information

**Supplementary Information:**

The online version contains supplementary material available at 10.1007/s00467-024-06316-6.

## Introduction

Glomerular filtration rate (GFR) is the best-known measurement of kidney function and reflects the ability of the kidney(s) to clear a particular substance from blood plasma [1]. There have been several methods developed to measure GFR. Kidney inulin clearance is considered the gold standard for measuring GFR. However, the technical difficulties with administering and measuring inulin, which include performing constant inulin infusions and collecting frequent timed urines, have made this impractical in clinical pediatric practice [2]. In children, measuring GFR from plasma clearance of iohexol is a reliable, well-tolerated method. Iohexol is a non-ionic, low osmolar, contrast medium that is safe and nontoxic [3, 4]. No serious adverse events were noted in more than 15 years of experience in Scandinavia, and in more than 900 GFR determinations performed in the NIH-sponsored study entitled Chronic Kidney Disease in Children (CKiD) [5, 6]. Additionally, there is excellent correlation between GFR values obtained with iohexol compared to inulin clearance and therefore, iohexol clearance is often used as a validated surrogate standard [3, 4, 7]. Routine clinical use, however, is not practical due to the need for several timed blood draws.

Because of these difficulties and limitations, serum creatinine is a commonly used marker to estimate GFR in the clinical setting. In the pediatric population, serum creatinine (Cr)-based GFR estimates are often determined using the Bedside Schwartz or U25 formula [6, 8]. Although Cr is a convenient and inexpensive marker, it is affected by non-kidney factors such as age, body mass, sex, medications, and non-kidney elimination [9]. Furthermore, Cr is secreted by the proximal tubules, which can overestimate GFR up to 10–20% [10]. Prior studies have investigated the use of cystatin C (CysC) as an alternative marker to measure kidney function as it is not confounded by factors seen with Cr. It is a non-glycosylated cysteine protease inhibitor protein that is produced at a constant rate in nearly every nucleated cell in the human body [11]. CysC is freely filtered through the glomerular membrane and is then reabsorbed and almost entirely catabolized in the proximal tubules; it is not secreted in the renal tubules or extrarenally eliminated [11]. The constancy of CysC production is independent of inflammatory conditions, muscle mass, sex, body composition, and age (after 12 months of age) [11]. Some studies suggest that steroids, diabetes mellitus with ketonuria, and thyroid dysfunction may influence serum CysC levels [12–14]. Very large doses of glucocorticoids have been described to increase the production of CysC, whereas low and medium doses do not seem to alter the production [12, 15, 16]. Several small studies have shown that the concentration of serum CysC is better correlated with GFR than serum Cr in children [17, 18]. Moreover, subtle decrements in GFR are more readily detected by changes in CysC than by Cr [18].

Accurate monitoring of estimated GFR (eGFR) after kidney transplantation is essential for early detection of allograft dysfunction, thus allowing for early intervention and prolonged graft survival. There are limited studies examining the performance of serum Cr- vs. CysC-based eGFR equations among pediatric kidney transplant patients, especially using the newer estimating equations such as the CKiD under 25 (U25) formulas in this group. If CysC correlates better with iohexol than creatinine in estimating GFR, it could be an attractive alternative or important adjunct for assessing allograft function in pediatric kidney transplant recipients.

We hypothesize that CysC-based equations perform better, as variables that can interfere with Cr values are less likely to occur with CysC. The goal of this study was to assess the performance of serum Cr- and CysC-based GFR estimating equations in reference to the validated measured iohexol GFR (mGFR). Secondary analysis evaluated the accuracy of these formulas among patients with evidence of histological change on kidney allograft biopsy and the ability to correctly classify chronic kidney disease (CKD) stage.

## Materials and methods

### Patient population

In this single-center study, serum Cr (enzymatic), CysC, and mGFR were measured among 45 kidney transplant recipients on the day they were seen for a protocol (6, 12, or 24 months post-transplant) or for-cause kidney transplant biopsy (increase in Cr, *de novo* human leukocyte antigen donor-specific antibodies (HLA-DSA), surveillance after treatment for rejection). Patients were induced with basiliximab or thymoglobulin and maintained on tacrolimus, mycophenolate mofetil, and steroid-free or steroid-based immunosuppression (prednisone or prednisolone 0.07–0.1 mg/kg/day, maximum of 5 mg daily). Low-dose trimethoprim-sulfamethoxazole (5–10 mg/kg/day trimethoprim) was used for *Pneumocystis jirovecii* pneumonia prophylaxis for the first 12 months post-transplant or 12 months after treatment for rejection. Patients were included in the study if they were between the age of 1–18 years old and kidney function was in steady state based on three separate serial determinations of serum Cr over a period of up to 6 months, as patients obtain labs no less frequently than every 3 months (interval dependent on patient’s duration post-transplant). In subjects with deviations from baseline Cr, the first Cr value did not differ more than 20% from the third and there was not a consistent upward or downward trend [19]. Patients were excluded if their kidney function was not in steady state, had known diabetes mellitus or thyroid dysfunction, had an allergy to iohexol or other contrast media, or family and/or patient did not consent to the study.

Biopsies were graded by the 2013 Banff criteria and reviewed by a pathologist who was blinded to each subject’s kidney function [20]. Luminex-based single-antigen bead assays (One Lambda Inc) were used to determine the antibody specificity and the mean fluorescence intensity (MFI). Antibodies were considered present when these intensity values were ≥ 1000 for HLA-A, -B, -DR, -DQ, and ≥ 2000 for HLA-C and -DP [21].

### Measurement of iohexol GFR

Two peripheral intravenous (IV) lines were placed—one for iohexol and maintenance IV fluid administration with normal saline (45 cc/hour up to 100 cc/hour; rate determined by the Holliday-Segar formula) and the second IV for serial blood draws [22]. After a patient’s biopsy was performed and patient was resting in the post-anesthesia recovery unit, IV fluids were initiated and 5 ml of iohexol (Omnipaque 300 supplied by Dr. Schwartz Lab, University of Rochester, 601 Elmwood Ave., Box 777, Room 2–5747, Rochester, NY 14642) was administered over 1–2 min. IV fluids were continued for the duration of the study. The blood (1 ml) was drawn for determination of iohexol levels at 10, 30, 120, and 300 min post-iohexol infusion [5, 6].

### Measurement of creatinine and cystatin C

Immediately post-biopsy, baseline serum enzymatic Cr and CysC (turbidimetric method, Gentian AS, Moss, Norway) levels were obtained at the beginning of the study and at the completion of the study at 300 min post-iohexol infusion. Cr and CysC were analyzed on the Olympus System and AU400 Olympus System, respectively, at the University of California’s Department of Pathology and Laboratory Medicine Outreach Laboratory, Los Angeles, CA. Among those who received a for-cause kidney transplant biopsy, Cr and CysC values were obtained prior to treatment based on biopsy findings. Therefore, no patients were on high-dose steroid therapy at the time of the study; if indicated, steroid treatment was given after their study day.

### Estimation of GFR

The following equations were used to estimate GFR (ml/min/1.73 m^2^). The Gentian CysC values utilized for all the equations below are standardized against the International Federation of Clinical Chemistry (IFCC) reference material. Because the Cr-CysC-based CKiD eGFR equation was developed before IFCC calibrated values, the Gentian CysC number was divided by 1.17 to provide a more accurate estimation of GFR [23].

1. Bedside Schwartz [6]

eGFR = 0.413 × (ht/Cr)

height (ht) = centimeters

Cr = mg/dl

2. CKiD under 25, serum creatinine (U25-Cr) [8]

eGFR = K × ht/Cr

K = sex- and age-dependent values

ht = meters

Cr = mg/dl

3. Gentian cystatin C (Gentian CysC) [24]

eGFR = 79.901 × CysC^−1.4389^

CysC = mg/l

4. CAPA equation (Caucasian, Asian, pediatric, adult) [25]

eGFR = 130 × CysC^−1.069^ × age^−0.117^ − 7

CysC = mg/l

Age = years

5. CKiD under 25, cystatin C (U25-CysC) [8]

eGFR = K × 1/CysC

K = sex- and age-dependent values

CysC = mg/l

6. Creatinine-cystatin C-based CKiD equation (CKiD Cr-CysC) [26]

eGFR = 39.8 × [ht/Cr]^0.456^ × [1.8/CysC]^0.418^ × [30/BUN]^0.079^ × [1.076^male^] [1.00^female^] × [ht/1.4]^0.179^

ht = meters

Cr = mg/dl

BUN (blood urea nitrogen) = mg/dl

CysC = mg/l

7. CKiD under 25, serum creatinine + cystatin C (U25 Cr-CysC) [8]

eGFR = (U25-Cr + U25-CysC)/2

### Statistical analysis

The performance of Cr and CysC eGFR were compared against mGFR using the Cr-based (Bedside Schwartz, U25-Cr), CysC-based (Gentian Cystatin C, CAPA), and combination Cr and CysC-based (CKiD Cr-CysC, U25 Cr-CysC) eGFR equations in terms of bias, precision, and accuracy [25, 27].

- Bias = eGFR − mGFR
- Precision = average bias ± 2SD of bias
- Accuracy = absolute percentage difference between eGFR and mGFR
  - P10 = the percentage of GFR estimates within 10% of mGFR
  - P30 = the percentage of GFR estimates within 30% of mGFR

Categorical variables were summarized by frequency and percentage and were compared across groups by the Fisher exact test. A two-sided 0.05 significance level was used throughout. Bland–Altman plots were conducted to evaluate the agreement between the eGFR equations and mGFR. CKD stage was assigned based on the Kidney Disease Outcomes Quality Initiative (K/DOQI) clinical practice guidelines and each eGFR was compared to their respective mGFR to assess misclassification [1].

## Results

Table 1 presents the demographics of the cohort’s 45 subjects. Median age at the time of biopsy and mGFR determination was 12.5 years (interquartile range 5.7–16.8 years), and at 12.0 months post-transplant (interquartile range 6.2–25.8 months). The study group consisted of 66.7% males, and 62.2% deceased donor kidney transplants. A majority of the cohort was on steroid-based immunosuppression (95.6%). Median time between first and third Cr measurement to determine study eligibility was 42 days (IQR 20–63 days) with median intra-patient Cr variability of 0.01 mg/dL (IQR 0–0.1 mg/dL).

**Table 1:** Patient demographics

*n*	45
Age at transplant, years	12.0 (5.5–16.0)
Age at time of study, years	12.5 (5.7–16.8)
Time post-transplant, months	12.0 (6.2–25.8)
Gender	
Female	15 (33.3)
Male	30 (66.7)
Transplant type	
Deceased-donor	28 (62.2)
Living-related or living-unrelated	17 (37.8)
Race	
African American	2 (4.4)
Hispanic	25 (55.6)
White	15 (33.3)
Asian	3 (6.7)
Original disease	
CAKUT	24 (53.3)
FSGS	4 (8.9)
Glomerulonephritis	9 (20.0)
Other	8 (17.8)
Steroid-based immunosuppression	43 (95.6)
Trimethoprim-sulfamethoxazole use*	24 (53.3)

Values are expressed as *n* (%) or median (IQR). *CAKUT*, congenital anomalies of the kidney and urinary tract; *FSGS*, focal segmental glomerulosclerosis

*Trimethoprim-sulfamethoxazole use for *Pneumocystis jiroveccii* pneumonia prophylaxis

Patient characteristics are highlighted in Table 2. A total of 45 mGFR measurements were performed among the 45 patients with a total of 315 eGFR assessments using the 7 eGFR equations; all subjects enrolled completed the study. Median mGFR was 93.3 ml/min/1.73 m^2^ (interquartile range 72.9–110.4 ml/min/1.73 m^2^) with 57.8% having mGFR $\ge$ 90 ml/min/1.73 m^2^, 33.3% between 60 and 89 ml/min/1.73 m^2^, and 8.9% between 30 and 59 ml/min/1.73 m^2^. There were no differences in the average mGFR between the 21 subjects without histological changes on biopsy vs. the 24 subjects with changes (92 vs. 90 ml/min/1.73 m^2^, respectively; *P* = 0.74). Twenty-nine subjects (64.4%) had a protocol biopsy performed. Among 16 patients (35.6%) who received a for-cause biopsy, 10 (22.2%) were for the development of *de novo* HLA-DSA. Among the 24 patients (53.3%) with histological changes on biopsy, 9 (20.0%) had interstitial fibrosis and tubular atrophy, 8 (17.8%) had isolated acute cellular rejection (ACR), 4 (8.9%) had isolated antibody-mediated rejection (ABMR), and 1 (2.2%) had mixed ACR and ABMR.

**Table 2:** Patient characteristics

*n*	45
Body weight, kg	30.8 (16.8–58.3)
Height, cm	132.8 (103.0–156.1)
BMI (kg/m^2^) percentile	71 (35.0–91.5)
Creatinine, mg/dl	0.7 (0.5–1.0)
Cystatin C, mg/dl	1.0 (0.8–1.2)
mGFR, ml/min/1.73 m^2^	93.3 (72.9–110.4)
mGFR, ml/min/1.73 m^2^	
< 30	0
30–59	4 (8.9)
60–89	15 (33.3)
≥ 90	26 (57.8)
Protocol biopsy	29 (64.4)
6 months	10 (22.2)
12 months	14 (31.1)
24 months	5 (11.1)
For-cause biopsy	16 (35.6)
Increase in creatinine	3 (6.7)
*De novo* HLA-DSA	10 (22.2)
Surveillance after rejection treatment	3 (6.7)
Histological changes on biopsy	24 (53.3)
IFTA	9 (20.0)
ACR	8 (17.8)
ABMR	4 (8.9)
ACR + ABMR	1 (2.2)
Other	2 (4.4)

Values are expressed as *n* (%) or median (IQR). *BMI*, body mass index; *mGFR*, measured glomerular filtration rate by iohexol clearance; *HLA-DSA*, human leukocyte antigen donor-specific antibodies; *IFTA*, interstitial fibrosis and tubular atrophy; *ACR*, acute cellular rejection; *ABMR*, antibody-mediated rejection

The bias, precision, and accuracy defined as percentage of estimates within 10% and 30% of mGFR (P10 and P30, respectively) for the estimating equations are presented in Table 3 for the whole cohort and in Table 4 for the subgroup of 24 individuals with histological changes on biopsy. The mean bias was small with the Gentian CysC formula at 0.1 ml/min/1.73 m^2^ (IQR − 16.6 to 12.9 ml/min/1.73 m^2^) in the entire cohort and − 5.6 ml/min/1.73 m^2^ (IQR − 19.3 to 0.6 ml/min/1.73 m^2^) in the subgroup with histological changes. There were no differences in the mean bias of eGFR equations in the presence or absence of low-dose trimethoprim-sulfamethoxazole use to prevent *Pneumocystis jirovecii* pneumonia (data not shown). Table 3 and Table 4 also highlight CysC-based equations U25-CysC, CKiD Cr-CysC, and U25 Cr-CysC had better precision with a smaller range in the 95% limits of agreement among the whole group and in those with changes on biopsy (61.0–62.0 vs. 78.2–98.6 ml/min/1.72 m^2^ and 60.2–62.4 vs. 78.0–82.8 ml/min/1.73 m^2^, respectively).

**Table 3:** Bias, precision, and accuracy of the eGFR equations compared to mGFR

Equation	Mean bias (IQR) (ml/min/1.73 m^2^)	Precision (ml/min/1.73 m^2^)	P10, *n* (%)	P30, *n* (%)	P (P10)	P (P30)
Bedside Schwartz	− 12.7 (− 22.4 to − 0.8)	− 51.8 to 26.4	11 (24.4)	36 (80.0)	0.18	0.38
U25-Cr	− 17.0 (− 26.9 to − 7.3)	− 58.2 to 24.2	7 (15.6)	36 (80.0)	0.02	0.38
Gentian CysC	0.1 (− 16.6 to 12.9)	− 46.8 to 47.0	17 (37.8)	37 (82.2)	> 0.99	0.55
CAPA	15.0 (− 0.6 to 30.1)	− 34.3 to 64.3	11 (24.4)	33 (73.3)	0.18	0.1
U25-CysC	− 6.0 (− 16.3 to 5.5)	− 36.5 to 24.5	17 (37.8)	40 (88.9)	> 0.99	Ref
CKiD Cr-CysC	− 10.4 (− 16.9 to − 2.1)	− 41.4 to 20.6	18 (40.0)	37 (82.2)	Ref	0.55
U25 Cr-CysC	− 11.5 (− 19.7 to − 3.8)	− 42.2 to 19.2	14 (31.1)	36 (80.0)	0.51	0.38

*IQR*, interquartile range; *eGFR*, estimated glomerular filtration rate; *mGFR*, measured glomerular filtration rate by iohexol clearance; *Cr*, creatinine; *CysC*, cystatin C. P10, the percentage of GFR estimates within 10% of mGFR; P30, the percentage of GFR estimates within 30% of mGFR; Ref, reference. Bias = eGFR-mGFR. Precision = average bias ± 2 standard deviation of (eGFR-mGFR). Accuracy is defined by the P10 and P30

**Table 4:** Bias, precision, and accuracy of the eGFR equations compared to mGFR in the presence of histological changes on allograft biopsy

Equation	Mean bias (IQR) (ml/min/1.73 m^2^)	Precision (ml/min/1.73 m^2^)	P10, *n* (%)	P30, *n* (%)	P (P10)	P (P30)
Bedside Schwartz	− 15.0 (-24.7 to − 2.9)	− 54.4 to 24.4	6 (25.0)	19 (79.2)	0.36	> 0.99
U25-Cr	− 19.7 (− 28.0 to − 9.6)	− 58.7 to 19.3	3 (12.5)	18 (75.0)	0.05	0.72
Gentian CysC	− 5.6 (− 19.3 to 0.6)	− 46.7 to 35.5	10 (41.7)	20 (83.3)	Ref	Ref
CAPA	11.1 (0.5 to 26.8)	− 30.3 to 52.5	7 (29.2)	20 (83.3)	0.55	> 0.99
U25-CysC	− 10.1 (− 20.9 to − 3.0)	− 40.2 to 20.0	10 (41.7)	20 (83.3)	> 0.99	> 0.99
CKiD Cr-CysC	− 13.6 (− 23.2 to − 5.7)	− 44.8 to 17.6	6 (25.0)	19 (79.2)	0.36	> 0.99
U25 Cr-CysC	− 14.9 (− 24.8 to − 7.5)	− 45.3 to 15.5	5 (20.8)	18 (75.0)	0.21	0.72

*eGFR*, estimated glomerular filtration rate; *mGFR*, measured glomerular filtration rate by iohexol clearance; *IQR*, interquartile range; *Cr*, creatinine; *CysC*, cystatin C. P10, the percentage of GFR estimates within 10% of mGFR; P30, the percentage of GFR estimates within 30% of mGFR; *Ref*, reference. Bias = eGFR-mGFR. Precision = average bias + 2 standard deviation of (eGFR-mGFR). Accuracy is defined by the P10 and P30

Among the whole cohort, U25-CysC had the higher accuracy with 88.9% of subjects within 30% of mGFR and 37.8% within 10% of mGFR (Table 3). P30 was otherwise essentially similar across all formulas assessed, with 70–80% of estimates within 30% of mGFR. While P10 was higher in CKiD Cr-CysC (40.0%), Gentian CysC and U25 Cr-CysC performed quite similarly with 37.8% of estimates within 10% mGFR, compared to the lowest P10 in U25-Cr (15.6%) and 24.2–31.1% in the remainder formulas. In the subgroup of subjects with histological changes on biopsy, U25-CysC and Gentian CysC had better accuracy with both having 83.3% of estimates within 30% of mGFR and 41.7% within 10% of mGFR (Table 4).

**Fig. 1:**
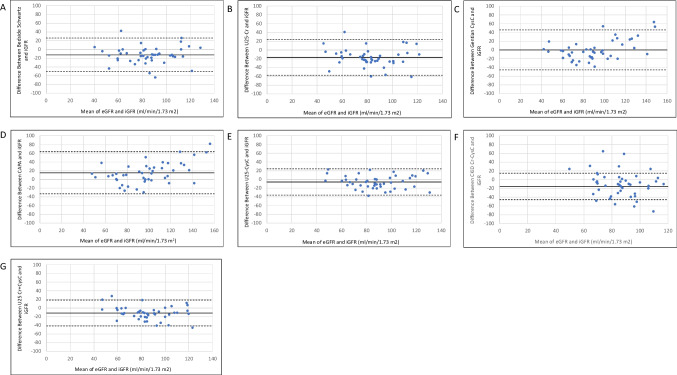
Bland–Altman plots showing the agreement between estimated GFR (eGFR) determined by (**A**) Bedside Schwartz; **B** U25-creatinine; **C** Gentian Cystatin C; **D** CAPA; **E** U25-Cystatin C; **F** CKiD creatinine-Cystatin C; **G** U25-Creatinine and Cystatin C equations and iohexol measured GFR (iGFR), plotted against the mean of the methods. The dashed lines highlight the limits of agreement between which 95% of the differences would be expected to fall. Solid line represents the mean bias, eGFR-iGFR

Bland–Altman analysis demonstrated the majority of GFR estimates were within the 95% limits of agreement, with U25-CysC, CKiD Cr-CysC, and U25 Cr-CysC having more narrow limits (Fig. 1). Bedside Schwartz, U25-Cr, U25-CysC, and U25 Cr-CysC formulas had relatively good precision in transplant patients with GFR between 60 and 100 ml/min/1.72 m^2^. Bedside Schwartz, Gentian CysC, CAPA, and U25-CysC estimating equations tended to overestimate GFR in those with a GFR > 100 ml/min/1.72 m^2^ (Fig. 1).

The ability of the equations to correctly classify CKD stage is shown in Table 5 for the whole cohort and in Table 6 for those with histological changes on biopsy. Misclassification of CKD stage ranged between 24.4 and 66.7% of patients within the two groups. Overall, the formulas misclassified CKD stages approximately 40–50% of the time, with the CAPA equation misclassifying CKD stage the least (24.4% of the time in the whole cohort and 33.3% of the time among the group with histological changes on biopsy). CAPA classified CKD G1 well, correctly classifying CKD G1 64.4% of the time. Among the whole cohort, CAPA statistically performed well in classifying CKD stage compared to the Bedside Schwartz, U25-Cr, and CKiD Cr-CysC (*P* = 0.05, 0.02, and 0.02, respectively). Within those who had changes on biopsy, CAPA statistically misclassified CKD stage less than CKiD Cr-CysC (*P* = 0.04). Collectively, the equations tended to underdiagnose CKD G1 and over-diagnose CKD G2 (Tables 5 and 6).

**Table 5:** CKD classification based on mGFR and eGFR

Equation	G1 (≥ 90 ml/min/1.73 m^2^)	G2 (60 to 89 ml/min/1.73 m^2^)	G3 (30 to 59 ml/min/1.73 m^2^)	G4 (15 to 29 ml/min/1.73 m^2^)	Misclassification of CKD stage in relation to iGFR	*P*
mGFR	26 (57.8)	15 (33.3)	4 (8.9)	0 (0.0)	-	–
Bedside Schwartz	12 (26.7)	21 (46.7)	12 (26.7)	0 (0.0)	21 (46.7)	0.05
U25-Cr	7 (15.6)	28 (62.2)	9 (20.0)	1 (2.2)	23 (51.1)	0.02
Gentian CysC	19 (42.2)	17 (37.8)	9 (20.0)	0 (0.0)	14 (31.1)	0.64
CAPA	29 (64.4)	14 (31.1)	2 (4.4)	0 (0.0)	11 (24.4)	Ref
U25-CysC	15 (33.3)	25 (55.6)	5 (11.1)	0 (0.0)	18 (40.0)	0.18
CKiD Cr-CysC	11 (24.4)	28 (62.2)	6 (13.3)	0 (0.0)	23 (51.1)	0.02
U25 Cr-CysC	12 (26.7)	25 (55.6)	8 (17.7)	0 (0.0)	17 (37.8)	0.25

Values are expressed as *n* (%). *CKD*, chronic kidney disease; *mGFR*, measured glomerular filtration rate by iohexol clearance; *eGFR*, estimated glomerular filtration rate; *Ref*, reference. No subjects had stage 5 CKD (eGFR < 15 ml/min/1.73 m^2^)

**Table 6:** CKD classification based on mGFR and eGFR in subjects with the presence of histological changes on allograft biopsy

Equation	G1 (≥ 90 ml/min/1.73 m^2^)	G2 (60 to 89 ml/min/1.73 m^2^)	G3 (30 to 59 ml/min/1.73 m^2^)	Misclassification of CKD stage in relation to mGFR	*P*
mGFR	15 (62.5)	7 (29.2)	2 (8.3)	-	–
Bedside Schwartz	5 (20.8)	11 (45.8)	8 (33.3)	13 (54.2)	0.24
U25-Cr	2 (8.3)	15 (62.5)	7 (29.2)	14 (58.3)	0.15
Gentian CysC	9 (37.5)	10 (41.7)	5 (20.8)	11 (45.8)	0.56
CAPA	16 (66.7)	7 (29.2)	1 (4.2)	8 (33.3)	Ref
U25-CysC	5 (20.8)	17 (70.8)	2 (8.3)	14 (58.3)	0.15
CKiD Cr-CysC	4 (16.7)	15 (62.5)	5 (20.8)	16 (66.7)	0.04
U25 Cr-CysC	4 (16.7)	15 (62.5)	5 (20.8)	12 (50.0)	0.38

Values are expressed as *n* (%). *CKD*, chronic kidney disease; *mGFR*, measured glomerular filtration rate by iohexol clearance; *eGFR*, estimated glomerular filtration rate; *Ref*, reference. No subjects had CKD G4 (eGFR 15 to 29 ml/min/1.73 m^2^) or G5 (eGFR < 15 ml/min/1.73 m^2^)

Sub-analysis comparing the equations by the three following groups instead of individual equations was also performed: Cr-based equations, CysC-based equations, and combined Cr and CysC-based equations (Supplemental Tables S1 and S2). Within the whole cohort and those with changes on histology, mean bias was smaller with CysC-based equations and the combined Cr and CysC-based equations were more precise. CysC-based and combined Cr and CysC-based formulas were more accurate in the whole cohort, and among patients with histological changes on biopsy, CysC-based equations had higher accuracy. However, P30 performance was similar among all three groups of equations. CysC-based formulas misclassified CKD stages the least and the pooled groups tended to underdiagnose CKD G1 and over-diagnose CKD G2 and G3 (Supplemental Tables S3 and S4).

## Discussion

Among our pediatric kidney transplant cohort with stable kidney function and with predominantly steroid-based immunosuppression, CysC-based formulas (CysC alone or in combination with Cr) may estimate GFR better than equations that are solely Cr-based. We also assessed the performance of these equations among those with histologic changes to determine if any of the equations can better identify those who have acute changes in kidney function and may need an allograft biopsy to determine the cause. Our findings show the Gentian CysC formula had a smaller mean bias among the whole cohort and in the subgroup with histologic changes in biopsy. CysC-based U25-CysC, CKiD Cr-CysC, and U25 Cr-CysC equations had higher precision in the whole group and among those with histological changes on biopsy. Therefore, CysC-based formulas (alone or in combination with Cr) appeared to perform as well or better in estimating GFR among those with and without allograft injury compared to equations solely utilizing Cr. The ability to precisely detect acute changes in kidney function in the transplant population will have implications on allograft longevity.

Several studies in adult kidney transplant recipients also found CysC-based equations performed better compared to Cr-based formulas [28–30]. Nonetheless, there were variable GFR approximations even between different CysC formulas within each study, which can be attributed to the lack of standardized CysC assays and measurement techniques [27, 29]. In our study, we attempted to provide more accurate estimation of GFR by using a CysC assay that was standardized against IFCC reference material and accounted for variations when using eGFR equations developed before IFCC calibrated CysC values.

In contrast to our findings, previous studies showed CysC-based equations were inferior to Cr-based or combined Cr- and CysC-based formulas. Among pediatric kidney transplant patients on low-dose steroids < 2.5 mg/m^2^ per day, de Souza et al. concluded CysC-based formulas did not perform better than Cr-based formulas, but rather the CKiD combined Cr and CysC formula performed the best for patients with a GFR < 90 ml/min/1.73 m^2^ [31]. In a cross-sectional study with 1139 adult kidney transplant recipients > 1 year post-transplant and the majority (86.9%) on low-dose corticosteroid (5 mg or less per day), Cr-based equations alone or in combination with CysC were preferred among adult kidney transplant recipients due to low bias and better accuracy compared to CysC only formulas, which was similar to the findings of Selistre et al. in pediatric kidney transplantation [27, 32]. It is unclear, however, if Selistre’s study subjects were on high-dose steroid therapy which could have affected CysC results. In our study, Bland–Altman plots showed that most Cr- and CysC-based equations have good precision for GFR between 60 and 100 ml/min/1.73 m^2^, with a tendency in some to overestimate among GFRs > 100 ml/min/1.73 m^2^. While other studies have demonstrated the Cr-based Bedside Schwartz equation to overestimate GFR in general, our current study showed that this more likely occurs at GFR > 100 ml/min/1.73 m^2^, where it is somewhat less relevant [31, 33].

Although the CysC-based CAPA equation misclassified CKD stage less in our small cohort, misclassification remains a common occurrence for all equations, generally occurring at least 20% of the time in other studies, compared to approximately 40–50% of the time in this study [33–35]. Furthermore, our study highlights the trend of underestimating CKD G1 and overestimating CKD G2. In a pediatric liver transplant cohort, equations utilizing both Cr and CysC misclassified CKD stage the least compared to highest misclassification with Cr-based formulas [34]. Among 198 adult kidney transplant recipients with stable kidney function, the CysC-based Filler estimating equation classified more patients into the correct CKD stage compared to Cr-based equations, with the Filler equation accurately classifying 76% of patients vs. 65% and 69% with Cr-based MDRD and Cockcroft-Gault formulas [35]. Westland et al. evaluated eGFR equations among 77 children with solitary functioning kidney, noting the least misclassification in CysC-based Zapitelli equation at 22%, while urine Cr-clearance had the highest misclassification at 44% [33]. Therefore, CysC-based equations may perform better in classifying CKD stage compared to Cr-based formulas not only in the transplant population, but also in those with CKD. This finding is not surprising, as CysC values are not affected by medications, diet, and muscle mass, which varies widely in the pre-transplant and post-transplant population.

It is important to note that our cohort had relatively well-preserved kidney function with a median GFR of 93.3 ml/min/1.73 m^2^, compared to many other studies with a mean or median GFR of around 60 ml/min/1.73 m^2^ [27, 28, 30–32]. Although the CAPA and CKiD Cr-CysC equations were validated in subjects with a wide range of measured GFR (< 30 to > 90 ml/min/1.73 m^2^), the U25 and Bedside Schwartz’s populations had mild–moderate chronic kidney disease, with median measured GFR of approximately 48 ml/min/1.73 m^2^ (IQR 34–64 ml/min/1.73 m^2^) and 41 ml/min/1.73 m^2^ (IQR 32–52 ml/min/1.73 m^2^), respectively [6, 8, 25, 26]. Based on these studies’ validation groups and our cohort’s collective range of GFR, CysC-based equations may perform better than those that are solely Cr based. Although our study population overall had higher measured GFR, the most current eGFR equation (U25) likely performed well in our cohort in terms of bias, precision, and accuracy because U25 estimates account for changes between sex, age, and height/serum Cr, or 1/CysC – factors believed to strengthen limitations in earlier formulas [8].

Like most of the transplant studies discussed, our patients were largely maintained on steroid-based immunosuppression. One reason for CysC-based equations (with or without Cr) potentially performing better in our cohort compared to solely Cr-based equations could be that CysC is not influenced by the use of low-dose steroids, which was also noted in other studies [16, 28, 30]. While very large doses of glucocorticoids have been described to increase the production of CysC, low and medium doses do not seem to alter the production [11, 12, 15, 16, 34, 36]. Risch et al. showed that 5–10 mg/day of steroid exposure led to higher CysC concentrations compared to those not on steroids among adult kidney transplant recipients and that the rise in CysC was dose dependent. Nonetheless, Risch’s study found that CysC was more accurate than Cr in identifying GFR < 60 ml/min/1.73 m^2^ [12]. In comparison, Cr concentrations are dependent on a multitude of factors including sex, age, race, nutritional state, and muscle mass [37]. Moreover, medications commonly used in transplantation can interfere with Cr levels. Steroids have a direct catabolic effect leading to lower muscle mass, and tubular secretion of Cr can be blocked by trimethoprim [37]. In this study, the use of low-dose trimethoprim-sulfamethoxazole did not appear to impact the Cr concentrations and thus, the GFR estimates. In contrast, in a retrospective study of 76 adult kidney transplant recipients, Yamanaga et al. concluded very low-dose trimethoprim-sulfamethoxazole for *Pneumocystis jirovecii* pneumonia prophylaxis reversibly increased Cr by 6% [38]. The cumulative impact of factors that determine Cr levels can certainly affect the utility of Cr in accurately assessing GFR in the pediatric kidney transplant population and thus supports the use of both Cr and CysC post-transplantation.

There were several limitations in our study, with one attributed to not assessing thyroid function [14, 39, 40]. A meta-analysis evaluating serum CysC levels in 1265 patients with thyroid disease and 894 controls revealed higher CysC levels among hyperthyroid subjects compared to lower CysC values in those with hypothyroidism. Furthermore, with treatment of the thyroid disease, CysC levels were notably affected, and therefore, the study concluded that serum CysC could be a marker for monitoring thyroid disease [40]. Additionally, our study had a small sample of 45 patients that only included those who underwent a protocol biopsy in the first 2 years post-transplant, or a for-cause biopsy, which may have introduced selection bias. Furthermore, with over half of our cohort being Hispanic and over 65% male, applicability to other demographics may be limited. The inherent nature of graft attenuation over time could mean that for some subjects in our study, kidney function may not have been in steady state even though their GFR remained within the allotted 20% deviation from prior creatinine measurements. A majority of our subjects had relatively well-preserved graft function, with 91.1% of the group with mGFR > 60 ml/min/1.73 m^2^ (57.8% of the population with mGFR > 90 ml/min/1.73 m^2^), therefore, reducing the ability to generalize this study’s findings to those with more advanced stages of CKD. Moreover, our cohort only included subjects 6 months and more post-transplant. As a result, we are unable to extrapolate these results to those at earlier stages post-transplant. Lastly, there was also variability between equations assessed and CysC assays used in our study in contrast to others, resulting in substantial heterogeneity in the performance of equations and difficulty conducting head-to-head comparisons. With such variation in the performance of eGFR equations, one could consider utilizing the same equation to longitudinally follow a patient’s allograft function over time. Pottel et al. found that over a follow-up time of 20 years among 417 adult kidney transplant patients, Cr-based estimating equations correctly predicted the trajectory of measured GFR (urinary clearance of inulin) in kidney transplant recipients; however, they lacked precision and accuracy [41]. Among our cohort, CysC-based equations (CysC alone or in combination with Cr) may better estimate GFR in pediatric kidney transplant recipients, including those with perceived stable allograft function exhibiting changes on biopsy. However, it is important to note that while CysC-based equations appear to perform better, the degree of difference may not be clinically significant. Our data, therefore, does not demonstratively show superiority of one biomarker over the other. Thus, the utilization of CysC could be tailored to the individual patient. For example, a person with reduced or high muscle mass, or on medications that could impact Cr may benefit from CysC assessment as an adjunct measurement of kidney function. In conclusion, our study supports the monitoring of both CysC and Cr post-transplant, which should be validated in future prospective, multicenter clinical trials.

### Supplementary Information

Below is the link to the electronic supplementary material.

Graphical abstract (PPTX 154 KB)

Supplementary file2 (DOCX 14 KB)

Supplementary file3 (DOCX 14 KB)

Supplementary file4 (DOCX 14 KB)

Supplementary file5 (DOCX 14 KB)

## Data Availability

The datasets generated during and/or analyzed during the current study are available from the corresponding author on reasonable request.

## Abbreviations

ACR: Acute cellular rejection
ABMR: Antibody-mediated rejection
CAPA: Caucasian, Asian, pediatric, adult estimating glomerular filtration rate equation
CKD: Chronic kidney disease
CKiD: Chronic Kidney Disease in Children
CKiD Cr-CysC: Creatinine- and cystatin C-based Chronic Kidney Disease in Children GFR equation
Cr: Serum creatinine
CysC: Serum cystatin C
DSA: Donor-specific antibodies
eGFR: Estimated glomerular filtration rate
GFR: Glomerular filtration rate
HLA: Human leukocyte antigen
Ht: Height
IFCC: International Federation of Clinical Chemistry
IV: Intravenous
K/DOQI: Kidney Disease Outcomes Quality Initiative
mGFR: Measured glomerular filtration rate by iohexol clearance
MDRD: Modification of diet in renal disease
P10: Percentage of GFR estimates within 10% of measured GFR
P30: Percentage of GFR estimates within 30% of measured GFR
SD: Standard deviation
U25: CKiD under 25
U25-Cr: CKiD under 25, serum creatinine-based GFR equation
U25-CysC: CKiD under 25, serum cystatin C-based GFR equation
U25 Cr-CysC: CKiD under 25, serum creatinine- and serum cystatin C-based GFR equation
