# Supplementary material for: Comparison of estimated GFR using cystatin C versus creatinine in pediatric kidney transplant recipients
Source: Pediatr Nephrol. 2024 Mar 1;39(7):2177–86. doi: 10.1007/s00467-024-06316-6 (PMC11147893; doi:10.1007/s00467-024-06316-6)
Supplement: Supplementary file 1 — Graphical abstract (PPTX 154 KB) [file 467_2024_6316_MOESM1_ESM.pptx]

## Slide 1
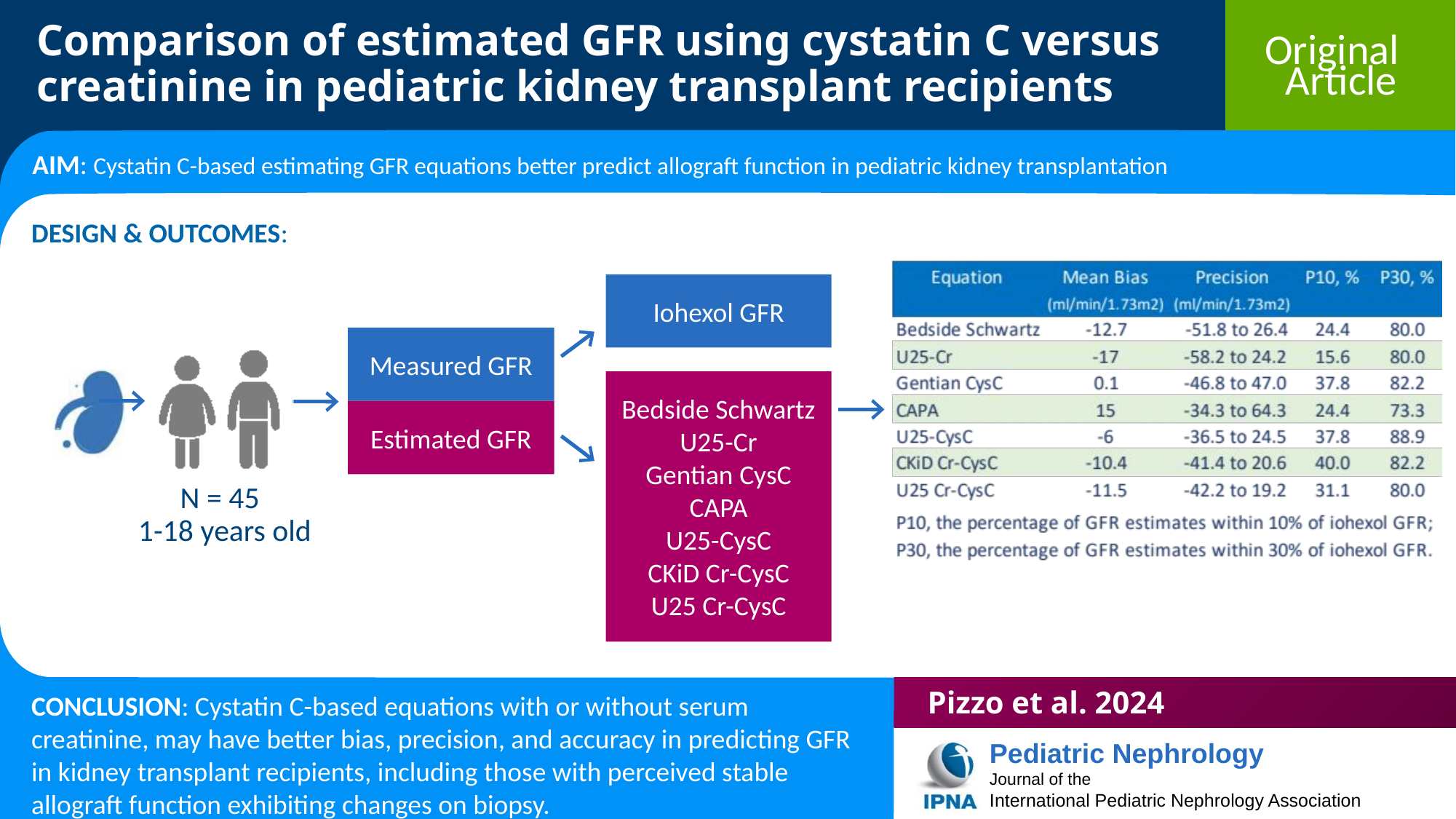

Comparison of estimated GFR using cystatin C versus creatinine in pediatric kidney transplant recipients
AIM: Cystatin C-based estimating GFR equations better predict allograft function in pediatric kidney transplantation
DESIGN & OUTCOMES:
Iohexol GFR
Measured GFR
Bedside Schwartz
U25-Cr
Gentian CysC
CAPA
U25-CysC
CKiD Cr-CysC
U25 Cr-CysC
Estimated GFR
N = 45
1-18 years old
Pizzo et al. 2024
CONCLUSION: Cystatin C-based equations with or without serum creatinine, may have better bias, precision, and accuracy in predicting GFR in kidney transplant recipients, including those with perceived stable allograft function exhibiting changes on biopsy.
