# Supplementary material for: Comparison of estimated GFR using cystatin C versus creatinine in pediatric kidney transplant recipients
Source: Pediatr Nephrol. 2024 Mar 1;39(7):2177–86. doi: 10.1007/s00467-024-06316-6 (PMC11147893; doi:10.1007/s00467-024-06316-6)
Supplement: Supplementary file 2 — Supplementary file2 (DOCX 14 KB) [file 467_2024_6316_MOESM2_ESM.docx]

| Table S1. Bias, precision, and accuracy of the eGFR equations compared to mGFR. | | | |  |  |  |
| --- | --- | --- | --- | --- | --- | --- |
| Equation | Mean Bias (IQR) (ml/min/1.73m2) | Precision (ml/min/1.73m2) | P10, n (%) | P30, n (%) | P (P10) | P (P30) |
| Cr-based | -14.8 (-24.9 to -3.7) | -55.1 to 25.3 | 18 (20.0) | 72 (80.0) | 0.03 | 0.86 |
| CysC-based | -3.0 (-15.8 to 12.5) | -43.2 to 49.2 | 45 (33.3) | 110 (81.5) | 0.78 | Ref |
| Combined Cr and CysC-based | -11.0 (-17.5 to 0.0) | -41.7 to 19.7 | 32 (35.5) | 73 (81.1) | Ref | >0.99 |
| IQR, interquartile range; eGFR, estimated glomerular filtration rate; mGFR, measured glomerular filtration rate by iohexol clearance; Cr, creatinine; | | | | | | |
| CysC, cystatin C. P10, the percentage of GFR estimates within 10% of mGFR; P30, the percentage of GFR estimates within 30% of mGFR. Ref, | | | | | | |
| reference. Bias = eGFR-mGFR. Precision = average bias +/- 2 standard deviation of (eGFR-mGFR). Accuracy is defined by the P10 and P30. Cr-based | | | | | | |
| equations (Bedside Schwartz, U25-Cr); CysC-based equations (Gentian CysC, CAPA, U25-CysC); Combined Cr and CysC-based equations | | | | | | |
| (CKiD Cr-CysC, U25 Cr-CysC). |  |  |  |  |  |  |
