# Supplementary material for: Comparison of estimated GFR using cystatin C versus creatinine in pediatric kidney transplant recipients
Source: Pediatr Nephrol. 2024 Mar 1;39(7):2177–86. doi: 10.1007/s00467-024-06316-6 (PMC11147893; doi:10.1007/s00467-024-06316-6)
Supplement: Supplementary file 3 — Supplementary file3 (DOCX 14 KB) [file 467_2024_6316_MOESM3_ESM.docx]

| Table S2. Bias, precision, and accuracy of the eGFR equations compared to mGFR in the presence of histological changes on allograft biopsy. | | | | | | |
| --- | --- | --- | --- | --- | --- | --- |
| Equation | Mean Bias (IQR) (ml/min/1.73m2) | Precision (ml/min/1.73m2) | P10, n (%) | P30, n (%) | P (P10) | P (P30) |
| Cr-based | -17.4 (-26.0 to -5.2) | -56.5 to 21.7 | 9 (10.0) | 37 (41.1) | 0.06 | 0.68 |
| CysC-based | -1.5 (-15.0 to 13.5) | -43.2 to 40.2 | 27 (20.0) | 60 (44.4) | Ref | Ref |
| Combined Cr and CysC-based | -14.3 (-21.4 to -5.7) | -45.1 to 16.5 | 11 (12.2) | 37 (41.1) | 0.15 | 0.68 |
| eGFR, estimated glomerular filtration rate; mGFR, measured glomerular filtration rate by iohexol clearance; IQR, interquartile range; Cr, creatinine; CysC, cystatin C. P10, the percentage of GFR estimates within 10% of mGFR; P30, the percentage of GFR estimates within 30% of mGFR; Ref, | | | | | | |
| reference. Bias = eGFR-mGFR. Precision = average bias +/- 2 standard deviation of (eGFR-mGFR). Accuracy is defined by the P10 and P30. | | | | | | |
|  | | | | | |  |
