# Supplementary material for: Comparison of estimated GFR using cystatin C versus creatinine in pediatric kidney transplant recipients
Source: Pediatr Nephrol. 2024 Mar 1;39(7):2177–86. doi: 10.1007/s00467-024-06316-6 (PMC11147893; doi:10.1007/s00467-024-06316-6)
Supplement: Supplementary file 4 — Supplementary file4 (DOCX 14 KB) [file 467_2024_6316_MOESM4_ESM.docx]

| Table S3. CKD classification based on mGFR and eGFR. | | |  |  |  |  |
| --- | --- | --- | --- | --- | --- | --- |
| Equation | G1 (≥ 90 ml/min/1.73m2) | G2 (60 to 89 ml/min/1.73m2) | G3 (30 to 59 ml/min/1.73m2) | G4 (15 to 29 ml/min/1.73m2) | Misclassification of CKD stage in relation to iGFR | P |
| mGFR | 26 (57.8) | 15 (33.3) | 4 (8.9) | 0 (0.0) | - | -- |
| Cr-based | 19 (21.1) | 49 (36.3) | 21 (23.3) | 1 (1.1) | 44 (48.9) | 0.01 |
| CysC-based | 63 (46.7) | 56 (41.5) | 16 (11.9) | 0 (0.0) | 43 (31.9) | Ref |
| Combined Cr and CysC-based | 23 (25.6) | 53 (58.9) | 14 (15.6) | 0 (0.0) | 40 (44.4) | 0.07 |
| Values are expressed as n (%). CKD, chronic kidney disease; mGFR, measured glomerular filtration rate by iohexol clearance; eGFR, estimated | | | | | | |
| glomerular filtration rate; Ref, reference. No subjects had stage 5 CKD (eGFR < 15 ml/min/1.73m2). | | | | |  |  |
