# Supplementary material for: Comparison of estimated GFR using cystatin C versus creatinine in pediatric kidney transplant recipients
Source: Pediatr Nephrol. 2024 Mar 1;39(7):2177–86. doi: 10.1007/s00467-024-06316-6 (PMC11147893; doi:10.1007/s00467-024-06316-6)
Supplement: Supplementary file 5 — Supplementary file5 (DOCX 14 KB) [file 467_2024_6316_MOESM5_ESM.docx]

| Table S4. CKD classification based on mGFR and eGFR in subjects with the presence of histological changes on allograft biopsy. | | | | |  |
| --- | --- | --- | --- | --- | --- |
| Equation | G1 (≥ 90 ml/min/1.73m2) | G2 (60 to 89 ml/min/1.73m2) | G3 (30 to 59 ml/min/1.73m2) | Misclassification of CKD stage in relation to mGFR | P |
| mGFR | 15 (62.5) | 7 (29.2) | 2 (8.3) | - | -- |
| Cr-based | 7 (7.8) | 26 (28.9) | 15 (16.7) | 27 (30.0) | 0.36 |
| CysC-based | 30 (22.2) | 34 (25.2) | 8 (5.9) | 33 (24.4) | Ref |
| Combined Cr and CysC-based | 8 (8.9) | 30 (33.3) | 10 (11.1) | 28 (31.1) | 0.29 |
| Values are expressed as n (%). CKD, chronic kidney disease; mGFR, measured glomerular filtration rate by iohexol clearance; | | | | | |
| eGFR, estimated glomerular filtration rate; Ref, reference. No subjects had CKD G4 (eGFR 15 to 29 ml/min/1.73m2) or | | | | | |
| G5 (eGFR < 15 ml/min/1.73m2). | |  |  |  |  |
